# Pilotprojekt zum „Praktikum der Berufsfelderkundung“ im Zahnmedizinstudium mit Fokus auf den Erwerb kommunikativer Kompetenzen

**DOI:** 10.1007/s00103-023-03792-3

**Published:** 2023-11-13

**Authors:** Uta Grote, Hans-Jürgen Wenz, Ariel-Salomon Gutman, Katrin Hertrampf

**Affiliations:** 1 Dekanat Geschäftsbereich Lehre, Medizinische Fakultät, Christian-Albrechts-Universität zu Kiel, Haus 11, Arnold-Heller-Str. 3, 24105 Kiel, Deutschland; 2 Klinik für Zahnärztliche Prothetik, Propädeutik und Werkstoffkunde, Universitätsklinikum Schleswig-Holstein, Campus Kiel, Kiel, Deutschland; 3 Klinik für Mund‑, Kiefer- und Gesichtschirurgie, Universitätsklinikum Schleswig-Holstein, Campus Kiel, Kiel, Deutschland

**Keywords:** Kommunikation, Zahnärztliche Approbationsordnung, Studierende, Zahnmedizin, Praktikum, Communication, Dental licensing regulations, Students, Dentistry, Internship

## Abstract

**Hintergrund:**

Die neue zahnärztliche Approbationsordnung sieht ein Berufsfeldpraktikum vor, um den Studierenden einen ersten umfassenden Einblick in ihr späteres Berufsfeld zu geben. Verortung, Umsetzung und Bewertung sind nicht festgelegt und können innerhalb der universitären Zahnmedizin oder in Kooperation mit zahnärztlichen Praxen erfolgen. Die Kieler Zahnklinik entschied, ein Konzept für eine 2‑wöchige Lehrveranstaltung, inklusive Praktikum, in niedergelassenen Praxen zu entwickeln und dabei Aspekte der ärztlichen Gesprächsführung verstärkt zu berücksichtigen. Auf Basis einer theoretischen Einführung und eines E‑Learning-Moduls soll dies der Einstieg in ein longitudinales Kommunikationscurriculum sein. Im Sommersemester 2021 wurde ein Pilotprojekt mit dem Ziel durchgeführt, die erforderliche Infrastruktur und inhaltliche Ausgestaltung der Veranstaltung zu entwickeln und mit Lehrinhalten zu kommunikativen Kompetenzen zu verknüpfen.

**Methoden:**

Es wurde eine kontrollierte Interventionsstudie (ein Messzeitpunkt) mit 21 Studierenden des 4. Semesters (2 Gruppen mit und ohne E‑Learning-Modul) sowie 20 zahnärztlichen Praxen durchgeführt. Die Studierenden erhielten vor dem einwöchigen Praktikum Dokumente zur Organisation, zum Ablauf und zur Dokumentation verschiedener Gesprächssituationen. Studierende und Praxen wurden nach Praktikumsende umfassend evaluiert.

**Ergebnisse:**

Studierende und Praxen waren mit der Organisation, den zur Verfügung gestellten Dokumenten und mit dem Ablauf zufrieden. Das E‑Learning-Modul wurde positiv aufgenommen. Das Format des Praktikumsberichts erwies sich als nicht praktikabel und wurde adaptiert.

**Diskussion:**

Die Projektziele konnten mit Erfolg erreicht werden. Die Ergebnisse fließen in die konkrete Gestaltung und Umsetzung der neuen curricularen Veranstaltung ab dem Sommersemester 2022 ein.

**Zusatzmaterial online:**

Zusätzliche Informationen sind in der Online-Version dieses Artikels (10.1007/s00103-023-03792-3) enthalten.

## Abstract (en)

**Background:**

The new dental licensing regulations provide for a professional field internship to give students an initial, comprehensive insight into their later professional field. The location, implementation and assessment are not specified and can take place within university dentistry or in cooperation with dental practices. The Kiel Dental Clinic decided to develop a concept for a two-week course, including practical training in private practices, and to take greater account of aspects of medical interviewing. Based on a theoretical introduction and an e‑learning module, this is to be the introduction to a longitudinal communication curriculum. In the summer semester of 2021, a pilot project was carried out with the aim of developing the necessary course infrastructure and content and linking it with teaching content on communicative competences.

**Methods:**

A controlled intervention study (one measurement point) was conducted with 21 fourth-semester students (two groups with and without e‑learning module) and 20 dental practices. The students received documents on the organisation, procedure and documentation of various interview situations before the one-week internship. Students and practices were comprehensively evaluated after the end of the internship.

**Results:**

Students and practices were satisfied with the organisation, the documents provided and the process. The e‑learning module was well received. The format of the internship report proved to be impractical and was adapted.

**Discussion:**

The project goals were successfully achieved. The results will be incorporated into the concrete design and implementation of the new curricular course starting summer semester 2022.

## Einleitung

In Deutschland wurde über Jahrzehnte nach der zahnmedizinischen Approbationsordnung von 1955 gelehrt. Diese wurde durch eine erste Novelle am 08.07.2019 abgelöst und pandemiebedingt verspätet mit Beginn des Wintersemesters 2021/2022 eingeführt [1]. Im Gegensatz zum Studium der Humanmedizin, bei dem Studierende im vorklinischen Abschnitt im Rahmen der Veranstaltung „Berufsfelderkundung“ ein halbtätiges Praktikum im niedergelassenen Bereich durchführen müssen [2], waren bis zur Novelle der zahnärztlichen Approbationsordnung Veranstaltungen dieser Art im Studiengang „Zahnmedizin“ nicht vorgesehen.

Die neue zahnärztliche Approbationsordnung sieht, neben einem verpflichtenden Pflegepraktikum im vorklinischen und einer Famulatur im klinischen Abschnitt, ein Berufsfeldpraktikum vor. Dies muss bis zur ersten staatlichen Prüfung nach dem 4. Semester absolviert werden. Dieses Praktikum soll Studierenden zu Beginn des Studiums einen ersten umfassenden Einblick in ihr späteres Berufsfeld geben.

Die Verortung, inhaltliche Umsetzung und die Bewertung dieses Praktikums sind durch die Approbationsordnung nicht festgelegt und können somit innerhalb der universitären Zahnmedizin oder in Kooperation mit zahnärztlichen Praxen durchgeführt werden [3]. Ausgehend von dieser Situation entschied die Kieler Zahnklinik ein Konzept in Kooperation mit zahnärztlichen Praxen zu entwickeln, in dem auch Aspekte der ärztlichen Gesprächsführung berücksichtigt werden sollten, da kommunikative Kompetenzen eine zentrale Bedeutung in der Berufsausübung haben.

Das erfolgreiche Gespräch zwischen Behandlerinnen und Behandlern und ihren Patientinnen und Patienten bildet die Basis für die nachfolgende Diagnostik und Therapie sowie für eine vertrauensvolle Beziehung zwischen beiden Seiten [4, 5]. Patientinnen und Patienten wollen heutzutage in Entscheidungsprozesse eingebunden werden und das Gefühl haben, ausführlich informiert zu werden, um eine aktive Rolle im Entscheidungsprozess übernehmen zu können [6]. Dieses veränderte Rollenverhältnis kann aber nur dann erfolgreich verwirklicht werden, wenn entsprechende Aspekte und Strategien der Gesprächsführung bei einem z. B. Erstgespräch berücksichtigt werden [7]. Dies setzt voraus, dass es entsprechend im Curriculum berücksichtigt wird.

Diese kommunikativen Kompetenzen sind in der neuen zahnärztlichen Approbationsordnung dezidiert in den Zielen der Ausbildung genannt und die entsprechenden Kenntnisse und Fertigkeiten auch in den mündlich-praktischen Prüfungen der verschiedenen zahnärztlichen Fächer im 3. Abschnitt der zahnärztlichen Prüfung nachzuweisen. Allerdings sind für die Umsetzung keine Lehrveranstaltungen ausgewiesen, die sich spezifisch mit den Aspekten der Kommunikation (im zahnärztlichen Umfeld) beschäftigen. Deshalb wurde am Standort Kiel beschlossen ein longitudinales Kommunikationscurriculum zu entwickeln, das in verschiedene Lehrveranstaltungen über die gesamte Studiendauer eingebettet sein soll. Entsprechende Lernziele sind im Nationalen Kompetenzbasierten Lernzielkatalog der Medizin und der Zahnmedizin ausgeführt [8, 9].

Die Implementierung dieser Thematik in Lehrveranstaltungen soll den Studierenden ermöglichen, schon zu Beginn des Studiums erste Aspekte eines differenzierteren Rollenverständnisses ihres gewählten Berufs sowie eine besser ausgeprägte Kommunikationskompetenz zu erlangen. Die Studierenden können so schon früh lernen, die Determinanten zahnärztlichen Handelns, wie z. B. der Erstkontakt und kulturelle sowie sprachliche Barrieren in der Patientenversorgung, in der Praxis zu reflektieren.

Ergänzend zu der Entwicklung dieser Lehrinhalte sollte auch die Verknüpfung von E‑Learning-Inhalten der Kommunikation mit anderen Lehrveranstaltungen gemäß dem „Blended Learning“ berücksichtigt werden [10, 11].

Im Sommersemester 2021 wurde ein Lehrprojekt im Sinne eines Pilotprojektes mit dem Ziel durchgeführt, die erforderliche Infrastruktur und inhaltliche Ausgestaltung des „Praktikums der Berufsfelderkundung“ zu entwickeln und mit den notwendigen Lehrinhalten zu kommunikativen Kompetenzen zu verknüpfen.

## Methoden

### Studiendesign

Es wurde eine kontrollierte Interventionsstudie mit einem Messzeitpunkt mit Studierenden der Zahnmedizin und niedergelassenen zahnärztlichen Kolleginnen und Kollegen nach Abschluss der Lehrveranstaltung durchgeführt.

### Das Praktikum der Berufsfelderkundung am Beispiel des Standortes Kiel

Das Praktikum wurde in der grundlegenden Berechnung des Curricularnormwertes (CNW) der Stiftung für Hochschulzulassung mit 3 Semesterwochenstunden, aber kapazitätsneutral angesetzt. Daraufhin wurde in Kiel die Entscheidung getroffen, dies als 2‑wöchiges Blockpraktikum in einer zahnärztlichen Praxis durchzuführen.

Einführend zum Praktikum wurden 2 Vorlesungen von je einer Semesterwochenstunde im 1. und 2. Semester geplant. Im ersten Semester fand die Vorlesung „Einführung in die Zahnheilkunde“ statt, mit einem ersten Einblick in die verschiedenen Fächer der Zahnheilkunde, einer Einführung in die Morphologie, Struktur und Funktion der Zähne und des orofazialen Systems sowie einem Schwerpunkt auf Kariesprävention und individuelle Mundhygiene. Im zweiten Semester folgte die Vorlesung mit Inhalten zu Selbstorganisation/Selbstmanagement, Einführung in das Gesundheitswesen, Hygiene und Mikrobiologie, Ethik, Kommunikation und Achtsamkeit. Nach der Vorlesung „Kommunikation“ sollten die Studierenden anhand eines E‑Learning-Moduls eine Einführung zur ärztlichen Gesprächsführung erhalten. Dieses war verpflichtend und musste vor Beginn des Praktikums erfolgreich absolviert werden. Das Praktikum schloss mit einem Praktikumsbericht ab.

### Studienpopulation und Rekrutierung

Die Studienpopulation setzte sich aus Studierenden des 4. Semesters des Studienganges „Zahnmedizin“ der Zahnklinik Kiel zusammen (*N* = 64). Voraussetzungen für die Teilnahme an der Studie waren die Zugehörigkeit zu diesem Fachsemester, ausreichende Kenntnisse der deutschen Sprache und eine schriftliche Einverständniserklärung.

Die Rekrutierung der Studierenden erfolgte zu Beginn des Sommersemesters 2021. Die Studierenden erhielten über den E‑Mail-Semesterverteiler eine ausführliche Information zu dem geplanten Projekt, verbunden mit der Bitte, sich bei Interesse bei dem angegebenen Mitglied der Arbeitsgruppe zu melden. Danach wurde für die interessierten Studierenden eine Veranstaltung im Hörsaal der Zahnklinik in Präsenz durchgeführt, in der nochmals ausführlich über das Lehrprojekt informiert wurde und auf auftretende Fragen eingegangen wurde. Die Teilnahme war freiwillig. Nach dem Einholen der Einwilligungserklärung wurden die teilnehmenden Studierenden gebeten, eine Verschwiegenheitserklärung und einen Fragebogen zu soziodemografischen Angaben auszufüllen.

Die Studierenden wurden darüber informiert, dass ergänzend zum Kennenlernen eines Praxisalltages ihre Aufgabe darin bestand, während ihres 5‑tägigen Praktikums verschiedene Kommunikationssituationen zu beobachten und am Ende des Praktikums zu 2 dieser Situationen je einen strukturierten schriftlichen Praktikumsbericht nach einer vorher besprochenen Vorlage (Beobachtungsbogen) abzugeben. Dieser Bogen zur Dokumentation verschiedener Gesprächsführungen in mehrfacher Ausfertigung und ein Leitfaden zur Erstellung des Praktikumsberichtes wurden ihnen ausgehändigt und sie wurden über die Versendung des Evaluationsbogens nach Praktikumsende informiert. Abschließend wurde mit jedem Studierenden das gewünschte Praktikumszeitfenster und der präferierte regionale Bereich einer Praxis abgestimmt.

Die Teilnehmerinnen und Teilnehmer aus der zahnärztlichen Kollegenschaft wurden im Rahmen einer Fortbildungsveranstaltung des Kreisvereines Kiel und über das bestehende Netzwerk der Arbeitsgruppe rekrutiert. Alle interessierten Zahnärztinnen und Zahnärzte wurden persönlich über die Studie informiert und um Teilnahme gebeten. Als Einschlusskriterium für die Praxis galt ein Standort innerhalb von Schleswig-Holstein. Nach mündlicher Zusage wurden den Zahnärztinnen und Zahnärzten postalisch die Projektinformation, die Einwilligungserklärung und der Fragebogen zu den soziodemografischen Angaben mit einem Freiumschlag für die Rücksendung zugesendet. Nach Erhalt der Einwilligung wurden jeweils die präferierten Praktikumszeiten abgefragt.

Das angestrebte Sample lag bei 20 Studierenden (Gesamtzahl *N* = 64 Studierende) und 20 zahnärztlichen Praxen innerhalb Schleswig-Holsteins.

### Studienablauf

Die Studierenden wurden in 2 Gruppen unterteilt. Die Einteilung ergab sich aus dem präferierten Zeitraum der Studierenden für das Praktikum. Die beiden Gruppen führten zeitlich nacheinander das Praktikum durch. Die zweite Gruppe erhielt das zusätzliche E‑Learning-Angebot über das Studierenden-Portal OpenOLAT, das am Smartphone und PC nutzbar war.

Nach der Rekrutierung beider Gruppen erfolgte die Zuordnung der Studierenden zu jeweils einer Praxis. Anschließend wurden die Studierenden und die Praxen über das Praktikumszeitfenster per E‑Mail informiert und um Bestätigung gebeten. Die Studierenden wurden aufgefordert persönlich mit der zugeordneten Praxis Kontakt aufzunehmen, um das weitere Vorgehen zu besprechen.

Die Praktika für die erste Gruppe begannen nach Ende des Sommersemesters 2021. Etwa 2 Wochen nach dem letzten durchgeführten Praktikum der ersten Gruppe begannen die Praktika der zweiten Gruppe. Die zweite Gruppe erhielt etwa 2 Wochen vor Beginn ihrer Praktika den Link zum E‑Learning-Modul. Dieses bestand aus 3 Input-Präsentationen zu Grundlagen der Gesprächsführung mit Videos zu verschiedenen Gesprächssituationen aus dem medizinischen Kontext. Die Lehrvideos wurden im Rahmen eines medizinischen Lehrprojektes zur Verbesserung kommunikativer Kompetenzen durch das Institut für Medizinische Psychologie und Soziologie des Universitätsklinikums Schleswig-Holstein, Kiel, erstellt und diesem Projekt zur Verfügung gestellt. In ihrer jeweiligen Praktikumswoche erhielten die Studierenden per E‑Mail das codierte Formblatt für den Praktikumsbericht und den codierten Evaluationsbogen. Die teilnehmenden Praxen erhielten in der Praktikumswoche die Vorlage für die Praktikumsbestätigung für die/den jeweilige/n Studierende/n und ebenfalls einen codierten Evaluationsbogen.

### Messinstrumente

Nach der Einwilligung wurden die Studierenden sowie die Zahnärztinnen und Zahnärzte gebeten einen kurzen Fragebogen zu verschiedenen soziodemografischen Variablen auszufüllen.

Zur Erfassung verschiedener Kommunikationssituationen, als Basis für den zu erstellenden Praktikumsbericht, erhielten die Studierenden den Beobachtungsbogen „Calgary-Cambridge Observation-Guide“ [12] zur Untersuchung der Student-Patienten-Kommunikation (siehe Onlinematerial „Beobachtungsbogen“). Dieser „Beobachtungsbogen“ mit insgesamt 38 Items sollte die Studierenden bei der Dokumentation der unterschiedlichen Gesprächssituationen während ihres Praktikums unterstützen. Der Bogen ist so aufgebaut, dass zu Beginn der Gesprächsanlass, mögliche beteiligte Personen, das Geschlecht und das Alter der zu untersuchenden Person festgehalten werden können. Dann folgen unterschiedliche Abschnitte wie Gesprächsbeginn (7 Items), 14 Items zu Informationsgewinn, unterteilt in die Bereiche „Erfassen der Anliegen/Probleme“ (9 Items) und „Verständnis der Patientenperspektive“ (5 Items), Strukturierung der Konsultation (4 Items), der Beziehungsaufbau mit 12 Items, gegliedert in „angemessenes nonverbales Verhalten“ (5 Items), Verbindungsaufbau (4 Items) und Einbeziehung der Patientin bzw. des Patienten (3 Items) und abschließend der Gesprächsabschluss mit 6 Items, inklusive eines Kommentarfeldes. Die Beantwortung erfolgt mittels einer 3‑stufigen Likert-Skala (Ja/teilweise/Nein).

Nach dem Praktikum wurden die Studierenden und die Zahnärztinnen und Zahnärzte aufgefordert jeweils unterschiedliche Evaluationsbögen auszufüllen. Die Evaluation der Studierenden umfasste verschiedene Erhebungsbögen. Unabhängig von der Zuordnung zur Gruppe ohne E‑Learning-Modul oder mit E‑Learning-Modul erhielten alle Studierenden dieselben Fragebögen.

Der erste Evaluationsbogen umfasste die Einschätzung des Praktikums in Bezug auf den Aufenthalt in der Praxis zu folgenden Bereichen „Atmosphäre“, „Hilfsbereitschaft der Zahnärztin und des Zahnarztes“, „Betreuung“, „Austausch und Beobachtungen in der Praxis“, „Absprache zwischen Universität und Praxis“ und „Praktikumsdauer“ (7 Items). Der zweite Evaluationsbogen bezog sich auf die zur Verfügung gestellten Unterstützungsmaterialien für die Erstellung des Praktikumsberichtes, bestehend aus 4 Items. Die Beantwortung aller Items erfolgte mittels 5‑stufiger Likert-Skala und der Antwortoption „keine Angabe“ (trifft voll und ganz zu, trifft eher zu, trifft weder noch zu, trifft eher nicht zu, trifft gar nicht zu).

Die Studierenden, die das E‑Learning-Modul durcharbeiten sollten, erhielten ergänzend einen dritten Fragenbogen bestehend aus 5 Items mit einer 5‑stufigen Likert-Skala und der Antwortoption „keine Angabe“ zur „Einschätzung der Inhalte des E‑Learning Moduls“, „Vorbereitung auf das Praktikum“ sowie zur „Wahrnehmung“, zum „Verständnis“ und zur „Reflexion von ärztlichen Gesprächssituationen“ (trifft voll und ganz zu, trifft eher zu, trifft weder noch zu, trifft eher nicht zu, trifft gar nicht zu).

Die teilnehmenden Zahnärztinnen und Zahnärzte wurden nach Beendigung des Praktikums gebeten einen Evaluationsbogen zu ihrer Einschätzung des Praktikums abzugeben. Er umfasste 6 Items im offenen Antwortformat zu „Informiertheit“, „Vorbereitung der Hospitation“, „Störung von Praxisabläufen“, „Akzeptanz der Studierenden“ sowie zu „Verbesserungsvorschlägen und Wiederaufnahme von Hospitierenden“. Durch Kategorisierung der Antworten wurden 3 Antwortoptionen generiert (Ja, Nein, keine Angabe).

Der abzugebende Praktikumsbericht war mit Bezug zum Beobachtungsbogen aufgebaut. Die Studierenden waren aufgefordert 2 konkrete Gesprächssituationen zu beschreiben. Abschließend sollten sie 3 Reflexionsfragen zur Gesprächssituation beantworten sowie anhand von 2 vertiefenden Fragen eine abschließende Bewertung abgeben und ein Fazit zu ihrem Praktikum ziehen. Der Umfang des Berichtes pro Gesprächssituation lag bei 4 vorgegebenen Seiten zur Darstellung der Gesprächssituation durch vorgegebene Felder zum Ankreuzen und 1–2 Seiten zur Reflexion der jeweiligen gewählten Situation.

### Statistische Auswertung

Die Studierenden und die Teilnehmerinnen und Teilnehmer aus den zahnärztlichen Praxen wurden pseudonymisiert. Die Praktikumsunterlagen und weitere Studienunterlagen wurden den Codes der jeweiligen Studierenden zugeordnet. Alle Analysen wurden mit SPSS 27.0 (SPSS Inc. Statistics for Windows, IBM, 2020, Armonk, NY, USA) durchgeführt. Deskriptive Statistiken werden in Tabellen dargestellt.

## Ergebnisse

Insgesamt nahmen 21 Studierende und 20 Praxen an dem Projekt teil (Tab. 1). 2 Studierende führten das Praktikum in unterschiedlichen Zeitfenstern (Juli und September) in derselben Praxis durch. Die Praktika wurden alle im Zeitfenster von Mitte Juli (Semesterende) und bis Ende September durchgeführt.

**Table 1:** 

Merkmale	Studierende *n* = 21	Zahnärztinnen und Zahnärzte *n* = 20
*Geschlecht*		
Weiblich *n* (%)	15 (71,4 %)	4 (20,0 %)
Männlich *n* (%)	6 (28,6 %)	16 (80,0 %)
*Alter in Jahren*		
Altersspanne	20–33 Jahre	41–74 Jahre
Mittelwert	25 Jahre	58 Jahre
*Erfahrungen zu Gesprächstechniken*		
Keine bekannt	5 (23,8 %)	**–**
Schule	6 (28,6 %)	**–**
Ausbildung	5 (23,8 %)	**–**
Studium	4 (19,0 %)	**–**
Sonstige	1 (4,8 %)	**–**
*Berufserfahrungen in Jahren*		
Spanne der Berufserfahrungen	**–**	12–42 Jahre
Mittelwert	**–**	28 Jahre

Von den 21 teilnehmenden Studierenden waren 15 Frauen und 6 Männer (71,4 % vs. 28,6 %). Dies entspricht der Geschlechterverteilung des gesamten Semesters. Das Durchschnittsalter der Studierenden lag bei 25 Jahren mit einer Altersspanne von 20 bis 33 Jahren.

Durch den in der Veranstaltung gesetzten Schwerpunkt der Arzt-Patienten-Kommunikation waren mögliche Vorerfahrungen zu Gesprächstechniken von besonderem Interesse. Insgesamt gaben 76,2 % der Studierenden (*n* = 16) an, dass ihre Erfahrungen aus der Schulzeit oder Ausbildungszeit stammen sowie aus dem Studium. 23,8 % der Studierenden (*n* = 5) hatten zuvor keine Erfahrungen gesammelt.

Von den teilnehmenden niedergelassenen Zahnärztinnen und Zahnärzten waren 16 Männer und 4 Frauen (80,0 % vs. 20 %) mit einem Durchschnittsalter von 58 Jahren bei einer Altersspanne von 41 bis 74 Jahren. Die Berufserfahrung lag bei 12 bis 42 Jahren. Die vollständige Ergebnisdarstellung für beide Gruppen ist in Tab. 1 zu finden.

### Evaluationen der Studierenden

Bei dem Vergleich der Einschätzungen der Studierenden zu ihrem Praktikum zeigte sich, dass sich die Erfahrungen der Studierenden insgesamt sehr ähnlich darstellten. Der Kontakt mit der Praxis und den Zahnärztinnen und Zahnärzten, der Einfluss der Praktikumszeit auf die eigene Entwicklung sowie die Absprachen zwischen der Universität und der Praxis im Vorfeld zur Organisation des Praktikums wurden von ihnen als sehr zufriedenstellend beschrieben. In der Einschätzung der Dauer des Berufsfeldpraktikums zeigen die Ergebnisse, dass die Studierenden ihr Praktikum eher als nicht zu kurz empfanden (*n* = 15; 71,4 %; trifft gar nicht zu/trifft eher nicht zu). Die ausführlichen Ergebnisse sind in Tab. 2 dargestellt.

**Table 2:** 

Items	Trifft voll und ganz zu	Trifft eher zu	Trifft weder noch zu	Trifft eher nicht zu	Trifft gar nicht zu	Keine Angabe
	+ +	+	+/−	−	− −	
	*n* (%)	*n* (%)	*n* (%)	*n* (%)	*n* (%)	*n* (%)
Die Atmosphäre in der Praxis war sehr angenehm	16 (76,2 %)	2 (9,5 %)	3 (14,3 %)	0	0	0
Ich habe den Zahnarzt, die Zahnärztin als sehr aufgeschlossen und hilfsbereit erlebt	19 (90,5 %)	2 (9,5 %)	0	0	0	0
Ich fühlte mich in der ausgewählten Praxis gut betreut	18 (85,7 %)	2 (9,5 %)	1 (4,8 %)	0	0	0
Der Austausch mit dem Zahnarzt war für meine eigene professionelle Weiterentwicklung hilfreich	15 (71,4 %)	4 (19,0 %)	2 (9,5 %)	0	0	0
Meine Beobachtungen in der Praxis sind für meine professionelle Weiterentwicklung hilfreich	13 (61,9 %)	7 (33,3 %)	1 (4,8 %)	0	0	0
Die Absprachen zwischen Universität und Praxis waren gut	8 (38,1 %)	8 (38,1 %)	3 (14,3 %)	1 (4,8 %)	0	1 (4,8 %)
Die Dauer des Praktikums war zu kurz	1 (4,8 %)	0	5 (23,8 %)	2 (9,5 %)	13 (61,9 %)	0

Im Weiteren wurde ausgewertet, wie der Leitfaden für die Erstellung des Praktikumsberichtes und der zu erstellende Praktikumsbericht von den Studierenden eingeschätzt wurden. Die Ergebnisse in der Tab. 3 weisen darauf hin, dass es den meisten Studierenden wichtig war, einen möglichst guten Bericht zu verfassen (*n* = 16; 76,2 %; trifft voll und ganz zu/trifft eher zu). Hinsichtlich der Unsicherheit, wie ein Praktikumsbericht aussehen soll, zeigen die Ergebnisse hingegen eher ein breites Spektrum. So schätzten insgesamt 33,3 % (*n* = 7) der Studierenden das Item: „Ich war unsicher wie der Praktikumsbericht aussehen sollte“, als voll und ganz zutreffend bzw. als eher zutreffend für sich ein. Hingegen traf für 23,8 % der Studierenden (*n* = 5) diese Aussage eher nicht oder gar nicht zu.

**Table 3:** 

Items	Trifft voll und ganz zu	Trifft eher zu	Trifft weder noch zu	Trifft eher nicht zu	Trifft gar nicht zu	Keine Angabe
	+ +	+	+/−	−	− −	
	*n* (%)	*n* (%)	*n* (%)	*n* (%)	*n* (%)	*n* (%)
Es war mir wichtig, einen möglichst guten Praktikumsbericht zu verfassen	14 (66,7 %)	2 (9,5 %)	2 (9,5 %)	3 (14,3 %)	0	0
Ich war unsicher, wie der Praktikumsbericht aussehen sollte	2 (9,5 %)	5 (23,8 %)	9 (42,9 %)	2 (9,5 %)	3 (14,3 %)	0
Ich fand die Anforderungen verständlich und eindeutig formuliert	7 (33,3 %)	6 (28,6 %)	6 (28,6 %)	2 (9,5 %)	0	0
Der Aufwand für die Anfertigung des Praktikumsberichts war zu hoch	0	1 (4,8 %)	7 (33,3 %)	7 (33,3 %)	6 (28,6 %)	0

Dagegen schienen aber die Anforderungen zum Praktikum für beide Gruppen mehrheitlich verständlich formuliert worden zu sein. Abschließend wurde auch der Aufwand für die Erstellung des Berichts eher als nicht zu hoch eingeschätzt.

### E-Learning

Bei der Betrachtung der Einschätzung des E‑Learning-Moduls für das Berufspraktikum zeigte sich ein überwiegend positives Bild (Tab. 4). Über die Hälfte der Studierenden äußerten sich positiv zum Einfluss des Moduls auf die Wahrnehmung von ärztlichen Gesprächssituationen in der Praxis (*n* = 6; 54,6 %; trifft voll und ganz zu/trifft eher zu). Ein geringerer Anteil der Studierenden gab an, dass ihnen das Modul beim Verstehen der verschiedenen Gesprächssituationen geholfen hat (*n* = 5; 45,5 %; trifft voll und ganz zu/trifft eher zu). Mehrheitlich positiv wurden von den Studierenden gesehen, dass der Inhalt des E‑Learning-Moduls nützlich für das Praktikum war und sie sich gut auf das Praktikum vorbereitet fühlten (*n* = 8; 72,8 %; trifft voll und ganz zu/trifft eher zu).

**Table 4:** 

Items	Trifft voll und ganz zu	Trifft eher zu	Trifft weder noch zu	Trifft eher nicht zu	Trifft gar nicht zu	Keine Angabe
	+ +	+	+/−	−	− −	
	*n* (%)	*n* (%)	*n* (%)	*n* (%)	*n* (%)	*n* (%)
Die Inhalte des E‑Learning-Moduls waren für mein Berufsfeldpraktikum nützlich	3 (27,3 %)	5 (45,5 %)	2 (18,2 %)	1 (9,1 %)	0	0
Das E‑Learning-Modul hat mir geholfen, die ärztl. Gesprächssituation in der Praxis wahrzunehmen	3 (27,3 %)	3 (27,3 %)	4 (36,4 %)	1 (9,1 %)	0	0
Das E‑Learning-Modul hat mir geholfen, die ärztl. Gesprächssituation in der Praxis zu verstehen	4 (36,4 %)	1 (9,1 %)	6 (54,5 %)	0	0	0
Das E‑Learning-Modul hat mir geholfen, die ärztl. Gesprächssituation in der Praxis zu reflektieren	4 (36,4 %)	3 (27,3 %)	4 (36,4 %)	0	0	0
Ich fühlte mich durch das E‑Learning-Modul für mein Praktikum gut vorbereitet	4 (36,4 %)	4 (36,4 %)	3 (27,3 %)	0	0	0

### Praktikumsbericht

Alle Praktikumsberichte zeigten eine nachvollziehbare und ausführliche Dokumentation der erlebten Arzt-Patienten-Gespräche in den Praxen. Die formalen Anforderungen wurden vollständig erfüllt. Dabei wurde der Fokus auf bedeutsame Momente in der Kommunikation zwischen Behandlerin und Behandler und ihren Patientinnen und Patienten gelegt. Das Spektrum reichte von Anamnesegesprächen, Behandlungsplanungen bis zur Aufklärung und Beratung. Insbesondere wurden die erschwerten Bedingungen, unter denen die Gespräche stattfanden, beleuchtet. Hierzu gehörten z. B. das Alter der Patientinnen und Patienten (Kinder oder Senioren), psychologische Umstände bei vorhandener Zahnarztangst sowie Verständigungsschwierigkeiten bei sprachlichen Barrieren.

Durch die fragengeleitete Dokumentation wurde der komplette Verlauf der Arzt-Patienten-Kommunikation in einzelnen Bausteinen abgebildet. In den Antworten auf die Reflexionsfragen haben sich die Studierenden sehr umfangreich mit der Rolle als Zahnärztin bzw. Zahnarzt in der Gesprächsführung auseinandergesetzt. Insgesamt wurden hierzu ungefähr 16.500 Wörter geschrieben. Hinzu kamen ausführliche Schilderungen der genauen Gesprächssituation, der Handlungsabläufe und der beteiligten Personen.

Die von den Studierenden aufgewendete durchschnittliche Arbeitszeit für die Erstellung des Berichts lag bei etwa 4,3 h, mit einer Spanne von 1–9 h.

### Evaluation der Zahnärztinnen und Zahnärzte

Die Evaluation der Zahnärztinnen und Zahnärzte erfolgte jeweils direkt nach Ende des Praktikums. Von den 20 teilnehmenden Zahnärztinnen und Zahnärzten in 20 Praxen fühlten sich 18 (90,0 %) gut informiert, 15 (75,0 %) gaben Mehraufwand an Arbeit durch die Vorbereitung auf das Praktikum an (Tab. 5). Insbesondere die Stellung von Praxiskleidung, die Organisation der Praktikumszeit, die Information des Praxisteams sowie die rechtliche Klärung hinsichtlich des Datenschutzes und die Hygieneeinweisung wurden in den Freitextfeldern als vorbereitende Aufgaben genannt. Darüber hinaus wurde dort ein Mehraufwand von 1–3 h angegeben.

**Table 5:** 

Items	Ja	Nein	Keine Angabe
	*n* (%)	*n* (%)	*n* (%)
Fühlten sich gut über das Berufsfeldpraktikum informiert	18 (90,0 %)	2 (10,0 %)	0
Haben Vorbereitungen vor Ankunft der Studierenden getroffen	15 (75,0 %)	5 (20,0 %)	1 (5 %)
Störungen von Praxisabläufen durch Studierende	1 (5,0 %)	19 (95,0 %)	0
Akzeptanz gegenüber den Studierenden durch Patientinnen und Patienten	19 (95,0 %)	0	1 (5,0 %)
Verbesserungsvorschläge für die Zukunft	15 (75,0 %)	4 (20,0 %)	1 (5,0 %)
Zukünftige Aufnahme von Praktikantinnen und Praktikanten vorstellbar	19 (95,0 %)	0	1 (5,0 %)

Wenn es um Praxisabläufe ging, konnten 19 (95,0 %) der Zahnärztinnen und Zahnärzte keine Störungen durch die Studierenden in ihrem Praxisalltag wahrnehmen (Tab. 5). Auch vonseiten der Patientinnen und Patienten schien eine hohe Akzeptanz der Studierenden vorzuliegen, 19 (95,0 %) der Zahnärztinnen und Zahnärzte bewerteten diese Frage positiv. Eine Praxis machte zu dieser Frage keine Angabe.

Verbesserungsvorschläge für die zukünftige Veranstaltung der Berufsfelderkundung wurden von 15 (75,0 %) der Zahnärztinnen und Zahnärzte genannt. Es wurde primär der Wunsch geäußert, die Berufsfelderkundung praxisorientierter auszurichten, damit die Studierenden bereits erste kleine Assistenztätigkeiten übernehmen können. In diesem Zusammenhang wurde auf die Klärung der Haftpflichtversicherung der Studierenden im Vorfeld hingewiesen.

Abschließend konnten sich 19 von 20 (95,0 %) Praxen eine Wiederaufnahme von Studierenden für das Berufsfeldpraktikum vorstellen. Eine Praxis konnte zu diesem Zeitpunkt noch keine Antwort dazu geben (Tab. 5).

## Diskussion

Da es sich bei Einführung des Praktikums der Berufsfelderkundung um eine gänzlich neue Veranstaltung im Studiengang „Zahnmedizin“ in Deutschland handelt und daher eine Diskussion von Erfahrungen nicht vorliegt, wird der Fokus der folgenden Diskussion auf den eigenen Ergebnissen und den sich daraus ergebenden Konsequenzen für die sich anschließende konkrete Umsetzung der Veranstaltung liegen.

Insgesamt zeigte sich in den Ergebnissen, dass die teilnehmenden Studierenden und die Zahnärztinnen und Zahnärzte sowohl mit der Organisation als auch mit der Durchführung des Praktikums zufrieden waren. Die Studierenden, die das E‑Learning absolviert haben, schätzten dieses positiv ein, sodass die Arbeitsgruppe entschied, ein solches Modul auch bei der Implementierung der Veranstaltung zu integrieren. Hier wurde dann aber das Konzept einer Arbeitsgruppe der Technischen Universität München präferiert. Diese Arbeitsgruppe hatte im Rahmen eines vom Bundesministerium für Bildung und Forschung (BMBF) geförderten Projektes verschiedene interaktive Module zur ärztlichen Gesprächsführung erfolgreich entwickelt. Im Rahmen einer Folgeförderung zur Umsetzung an anderen Standorten mit einer Begleitevaluation wurde gemeinsam konkret geplant dieses E‑Learning-Konzept am Kieler Standort einzusetzen und zu evaluieren.

Die Rekrutierung der Praxen und die Zuordnung der Studierenden erwiesen sich innerhalb des Projektes als durchaus zeitintensiv. Die Praktika selber liefen völlig unproblematisch ab, auch die von der Arbeitsgruppe vorgeschlagene Vorlage zur Bestätigung des Praktikums durch die Praxen erwies sich als praktikabel und wurde in das Portefeuille der Unterlagen für die Veranstaltung mit aufgenommen.

Die Praktikumsberichte der Studierenden waren insgesamt sehr gut ausgeführt. Die Berichte selber unterschieden sich in der Ausführlichkeit der Kommentierung der jeweiligen Gesprächssituationen. Insgesamt zeigte sich aber, dass durch die mehrseitige Dokumentation der beiden Situationen mit den abschließenden offenen Fragestellungen und der Kommentaroption viel zu umfangreiche Berichte entstanden waren und sich dieses Format somit für ein vollständiges Semester mit 65 Studierenden sowohl in der Benotung als auch der Rückmeldung an die Studierenden als nicht praktikabel erwies.

Nach Diskussion innerhalb der Arbeitsgruppe wurde empfohlen, dass die beiden zu dokumentierenden Gesprächssituationen während des Praktikums deutlich kürzer über festgelegte Fragen dargestellt werden sollten. Für jede Situation sollen die Studierenden abschließend eine kurze Reflexion mit maximal 50 Wörtern anfertigen. Der Leistungsnachweis soll sich dann auf „bestanden“ oder „nicht bestanden“ beschränken.

Nach Abschluss des Projektes wurden die Ergebnisse den teilnehmenden Zahnärztinnen und Zahnärzten in einer Videokonferenz vorgestellt und diskutiert. Mit den teilnehmenden Studierenden wurden die Ergebnisvorstellung und die Diskussion im Hörsaal der Zahnklinik durchgeführt. Danach folgte die konkrete Umsetzung bzw. Adaption für die erste Veranstaltung zum Sommersemester 2022.

Die regionale Zahnärztekammer Schleswig-Holstein bot Unterstützung bei der Zusammenstellung interessierter regionaler Praxen an. Gemeinsam mit der Arbeitsgruppe wurde ein Artikel zu dem Lehrprojekt im monatlichen regionalen Zahnärzteblatt publiziert. Dieser beinhaltete einen Aufruf an interessierte Praxen, sich bei der Kammer zu melden und sich in eine Praxisliste aufnehmen zu lassen. Diese Liste wird den Studierenden bei einer Praxissuche zur Verfügung gestellt. Die Aktualisierung und Pflege dieser Liste erfolgt durch die Kammer.

Die verschiedenen Unterstützungsmaterialien, wie die Checkliste zur Organisation und Durchführung des Praktikums, der Beobachtungsbogen und der Leitfaden zur Erstellung des Praktikumsberichtes, erwiesen sich für die Studierenden als hilfreich und unterstützend. Nach Abschluss des Projektes wurde die Checkliste durch die Anregungen der teilnehmenden Zahnärztinnen und Zahnärzte ergänzt, der Link zur regionalen Praxisliste integriert und die Empfehlungen für eine Berufshaftpflicht und die Hepatitis-B-Impfung mit aufgenommen.

Der Einsatz des Beobachtungsbogens sowohl zur Dokumentation von verschiedenen Gesprächssituationen in den Praxen als auch als unterstützendes Instrument für den zu erstellenden Praktikumsbericht erwies sich als geeignet und es wurde von der Arbeitsgruppe entschieden, dieses Instrument bei der Implementierung der Veranstaltung mit zu berücksichtigen. Der Leitfaden für den Praktikumsbericht wurde entsprechend den neuen Vorgaben adaptiert.

Somit wurde nach den Empfehlungen der Arbeitsgruppe in Abstimmung mit der Steuerungsgruppe folgender inhaltlicher und zeitlicher Ablauf der Veranstaltung „Praktikum der Berufsfelderkundung“ festgelegt.

- Die Vorlesung im ersten Semester bleibt unverändert.
- Die Vorlesung im zweiten Semester setzt sich aus den folgenden Themen zusammen: Selbstorganisation/Selbstmanagement (mit anschließendem Wahlfach zu dieser Thematik), Einführung in das Gesundheitswesen, kommunikative Kompetenzen, Ethik, Mikrobiologie/Hygiene und Achtsamkeit. Sie schließt mit einer Klausur ab.
- Nach der Veranstaltung zu kommunikativen Kompetenzen wird das interaktive E‑Learning-Modul für die Studierenden freigeschaltet. Dieses Modul muss bis zum Beginn des Praktikums in einer niedergelassenen Praxis erfolgreich abgeschlossen sein.
- Im Rahmen des Praktikums müssen verschiedene Gesprächssituationen dokumentiert werden. 2 Situationen müssen im Praktikumsbericht erläutert werden.
- Der Leistungsnachweis setzt sich aus der bestandenen Klausur, dem Zertifikat für das E‑Learning-Modul und dem durchgeführten Praktikum mit dem Praktikumsbericht zusammen.

Auf diese Veranstaltung aufbauend soll dann das longitudinale Curriculum zu kommunikativen Kompetenzen schrittweise in den darauffolgenden Semestern weiterentwickelt werden.

### Limitationen

Bei der Interpretation der Ergebnisse muss berücksichtigt werden, dass es sich um ein monozentrisches Projekt gehandelt hat. Darüber hinaus ist nicht auszuschließen, dass ein Selektionsbias bei den teilnehmenden Studierenden und Zahnärztinnen und Zahnärzte bestand.

## Fazit

Es konnte gezeigt werden, dass die Projektziele mit Erfolg erreicht werden konnten und die Ergebnisse unmittelbar in die konkrete Gestaltung und Umsetzung dieser neuen curricularen Veranstaltung ab dem Sommersemester 2022 einfließen und damit in den Stundenplan des Studienganges „Zahnmedizin“ fest verankert werden.

### Supplementary Information




