# Supplementary material for: Pilot project for the “Internship to Gain Experience in the Professional Field” in dentistry studies with a focus on acquiring communicative skills
Source: Bundesgesundheitsblatt Gesundheitsforschung Gesundheitsschutz. 2023 Nov 13;66(12):1380–8. [Article in German] doi: 10.1007/s00103-023-03792-3 (PMC10667377; doi:10.1007/s00103-023-03792-3)
Supplement: Supplementary file 1 [file 103_2023_3792_MOESM1_ESM.pdf]

Beobachtungsbogen[1] zum Erfassen von Gesprächsstrukturen
Eine Dokumentationshilfe

Dieser Beobachtungsbogen soll Sie dabei unterstützen, 3–5 unterschiedliche Gesprächssituationen in Ihrer Praktikumspraxis kurz festzuhalten. Kopieren Sie sich diesen Bogen mehrfach und tragen Sie Ihre Beobachtungen für die einzelnen Gespräche ein. Für Ihren Praktikumsbericht wählen Sie dann zwei Gesprächssituationen aus, die Sie in der Ihnen zugegangenen Formatvorlage für den Bericht ausführlicher darstellen wollen.

Gesprächsanlass

Beteiligte Personen

Geschlecht

Alter

Gesprächsbeginn

Die Behandelnde / Der Behandelnde …

	Ja	teilweise	nein
Kontaktaufnahme			
1. begrüßt mit Handschlag und redet mit Namen an bzw. fragt danach.	☐	☐	☐
2. stellt sich mit Namen und Funktion vor.	☐	☐	☐
3. zeigt Interesse und Respekt und sorgt dafür, dass der Patient bequem sitzt bzw. liegt.	☐	☐	☐
Gründe für die Konsultation			
4. erfasst nach einer offenen Frage die Probleme oder Anliegen.	☐	☐	☐
5. unterbricht zu Beginn nicht und hört aufmerksam zu.	☐	☐	☐
6. vergewissert sich, dass die Schilderung korrekt verstanden wurde und erfragt weitere Probleme.	☐	☐	☐
7. klärt gemeinsam mit dem Patienten den Gesprächsablauf und den zeitlichen Rahmen.	☐	☐	☐

[1] Es handelt sich um den Beobachtungsbogen „Calgary-Cambridge Observation Guide zur Untersuchung der Student - Patienten – Kommunikation“ [12].

Informationsgewinn

Die Behandelnde / Der Behandelnde …

Erfassen der Anliegen / Probleme	**Ja**	**teilweise**	**nein**
8. ermutigt den Patienten, seine Geschichte in eigenen Worten zu schildern	☐	☐	☐
9. verwendet situationsabhängig zuerst offene und im weiteren Verlauf auch geschlossene Fragen.	☐	☐	☐
10. hört aufmerksam zu, lässt aussprechen und erlaubt Pausen zum Nachdenken vor der Antwort.	☐	☐	☐
11. unterstützt den Patienten, verbal und nonverbal etwas auszudrücken (z.B. Wiederholung, Paraphrasierung, Kopfnicken, Schweigen)	☐	☐	☐
12. greift verbale und nonverbale Hinweise auf (z.B. Körpersprache, Mimik) und geht darauf ein.	☐	☐	☐
13. klärt Äußerungen, die unklar sind oder näher betrachtet werden sollten.	☐	☐	☐
14. fasst regelmäßig zusammen, um das Verständnis zu sichern und ermuntert zu korrigieren und zu ergänzen.	☐	☐	☐
15. drückt sich angemessen einfach und verständlich aus und vermeidet bzw. erklärt Fachsprache.	☐	☐	☐
16. klärt die Daten und den Ablauf der Ereignisse.	☐	☐	☐

Verständnis der Patientenperspektive	**Ja**	**teilweise**	**Nein**
17. erfasst aktiv und erkundet angemessen:			
a. Vorstellungen des Patienten (z.B. Ursachen)	☐	☐	☐
b. Sorgen des Patienten hinsichtlich der Probleme.	☐	☐	☐
c. Erwartungen (z.B. Ziele, erwartete Hilfe)	☐	☐	☐
d. Auswirkungen auf den Alltag des Patienten.	☐	☐	☐
18. ermutigt den Patienten Gefühle auszudrücken.	☐	☐	☐

Strukturierung der Konsultation

Die Behandelnde / Der Behandelnde…

Gesprächsorganisation	**Ja**	**teilweise**	**Nein**
19. fasst am Ende eines Themas zusammen, um das Verständnis zu bestätigen und zu sichern, dass nichts Wichtiges vergessen wurde; ermuntert den Patienten zu korrigieren.	☐	☐	☐
20. verdeutlicht und begründet den Übergang zu einem anderen Thema oder Gesprächsabschnitt.	☐	☐	☐
Unterstützung des Gesprächsfortschritts			
21. strukturiert das Interview in einem logischen Ablauf.	☐	☐	☐
22. steuert das Interview zeitlich und behält die Ziele im Auge.	☐	☐	☐

Beziehungsaufbau

Die Behandelnde / Der Behandelnde…

Angemessenes nonverbales Verhalten	**Ja**	**teilweise**	**Nein**
23. zeigt angemessenes nonverbales Verhalten			
a. Augenkontakt, Gesichtsausdruck.	☐	☐	☐
b. Körper- und Sitzhaltung, Gesten und Bewegungen.	☐	☐	☐
c. Sprache (Geschwindigkeit, Tonfall, Lautstärke, Tonlage)	☐	☐	☐
24. liest, schreibt bzw. verwendet den PC falls notwendig, so, dass der Dialog und die Beziehung nicht beeinträchtigt werden.	☐	☐	☐
25. zeigt der Situation angemessene Zuversicht.	☐	☐	☐
Verbindungsaufbau			
26. achtet die Rechtmäßigkeit der Patientenmeinung und -gefühle und wertet sie nicht.	☐	☐	☐
27. nimmt empathisch, verständnisvoll und wertschätzend die Meinung und Gefühle des Patienten auf.	☐	☐	☐
28. drückt Besorgnis, Verständnis sowie den Willen partnerschaftlich zu helfen aus und erkennt die Bewältigungsstrategien und Eigenverantwortung an.	☐	☐	☐
29. geht angemessen mit peinlichen oder beunruhigenden Themen und körperlichen Schmerzen um.	☐	☐	☐

Einbeziehung des Patienten			
30. teilt eigene Überlegungen mit und bezieht den Patienten mit ein.	☐	☐	☐
31. erläutert Fragen und Teile des Untersuchungsablaufs, die den Patienten überraschen könnten.	☐	☐	☐
32. bittet vorher um Erlaubnis und erklärt während der Untersuchung den Ablauf.	☐	☐	☐

Gesprächsabschluss

Die Behandelnde / Der Behandelnde…	Ja	teilweise	Nein
33. erklärt in angemessenen Zeitabständen und vermeidet Ratschläge, neue Informationen oder voreilige Meinungen.	☐	☐	☐
34. gibt alle vorläufigen Informationen strukturiert ohne den Patienten zu überfordern; vermeidet oder erklärt Fachsprache.	☐	☐	☐
35. verständigt sich mit dem Patienten über die nächsten Behandlungsschritte.	☐	☐	☐
36. überprüft das Verständnis des Patienten und ob dieser die Erläuterungen und Pläne akzeptiert. Versichert sich, dass alle Anliegen angesprochen wurden.	☐	☐	☐
37. fasst die Konsultation kurz zusammen.	☐	☐	☐
38. fragt den Patienten, ob noch etwas zu besprechen ist und bietet Raum Ausstehendes zu diskutieren.	☐	☐	☐

Zusatzbemerkungen:

....

....

....

....

....

....

....

....

....

....

....
